# Supplementary material for: MetaRibo-Seq measures translation in microbiomes
Source: Nat Commun. 2020 Jun 29;11:3268. doi: 10.1038/s41467-020-17081-z (PMC7324362; doi:10.1038/s41467-020-17081-z)
Supplement: Supplementary file 10 — Supplementary Data 7 [file 41467_2020_17081_MOESM10_ESM.zip › File2/Confidence_High_Taxonomy/320325_out.krona.html]

Javascript must be enabled to view this page.

members
magnitude
magnitudeUnassigned
count
unassigned
taxon
rank

320325\_out

120

2
superkingdom
120

976
120
phylum

class
120
200643

171549
order
120

family
119
815

816
genus
119

2292919
5
species

SRS013476\_contig\_number\_15301SRS024132\_contig\_number\_42518SRS024331\_contig\_number\_12917SRS046717\_contig\_number\_7008SRS078419\_contig\_number\_8269

species
1
671267

SRS893373\_contig\_number\_15467


SRS012849\_contig\_number\_30158SRS012969\_contig\_number\_27136SRS013098\_contig\_number\_39364SRS014412\_contig\_number\_21164SRS014459\_contig\_number\_33929SRS014534\_contig\_number\_1253SRS014923\_contig\_number\_28756SRS015065\_contig\_number\_33090SRS015578\_contig\_number\_28470SRS015663\_contig\_number\_18538SRS015694\_contig\_number\_20923SRS015794\_contig\_number\_12327SRS015890\_contig\_number\_9770SRS016335\_contig\_number\_48824SRS016495\_contig\_number\_16979SRS016897\_contig\_number\_contig-100\_38.25736SRS017859\_contig\_number\_163SRS018133\_contig\_number\_9620SRS018817\_contig\_number\_10578SRS018984\_contig\_number\_2983SRS019445\_contig\_number\_26181SRS019601\_contig\_number\_29365SRS019693\_contig\_number\_7235SRS019968\_contig\_number\_1669SRS020233\_contig\_number\_34313SRS020508\_contig\_number\_contig-100\_34.17772SRS020869\_contig\_number\_contig-100\_123.225073SRS022137\_contig\_number\_19909SRS043411\_contig\_number\_11140SRS044535\_contig\_number\_12356SRS045713\_contig\_number\_14631SRS046369\_contig\_number\_11362SRS047014\_contig\_number\_contig-100\_137.180337SRS047433\_contig\_number\_3933SRS048164\_contig\_number\_13990SRS048870\_contig\_number\_21027SRS049773\_contig\_number\_contig-100\_148.210189SRS050941\_contig\_number\_5300SRS051031\_contig\_number\_23558SRS052576\_contig\_number\_contig-100\_6.15515SRS053087\_contig\_number\_2911SRS053214\_contig\_number\_3871SRS053356\_contig\_number\_47566SRS053398\_contig\_number\_6014SRS054352\_contig\_number\_24257SRS054590\_contig\_number\_793SRS055982\_contig\_number\_12221SRS056273\_contig\_number\_14497SRS058070\_contig\_number\_11390SRS058770\_contig\_number\_14456SRS062654\_contig\_number\_533SRS063190\_contig\_number\_12886SRS065504\_contig\_number\_23413SRS075341\_contig\_number\_20367SRS075878\_contig\_number\_19936SRS076756\_contig\_number\_20352SRS076876\_contig\_number\_799SRS076976\_contig\_number\_contig-100\_2333.91333SRS077086\_contig\_number\_4915SRS077127\_contig\_number\_3591SRS077392\_contig\_number\_42495SRS077589\_contig\_number\_2551SRS077730\_contig\_number\_4492SRS077849\_contig\_number\_22376SRS098644\_contig\_number\_41479SRS098881\_contig\_number\_20138SRS100907\_contig\_number\_7200SRS101433\_contig\_number\_13424SRS103987\_contig\_number\_2468SRS103987\_contig\_number\_34172SRS104084\_contig\_number\_5390SRS1041138\_contig\_number\_12835SRS104975\_contig\_number\_contig-100\_406.126170SRS105153\_contig\_number\_39693SRS1054928\_contig\_number\_6485SRS1055049\_contig\_number\_9794SRS142599\_contig\_number\_31423SRS142712\_contig\_number\_3018SRS142890\_contig\_number\_40641SRS142980\_contig\_number\_6724SRS143181\_contig\_number\_15796SRS143417\_contig\_number\_33638SRS144537\_contig\_number\_38558SRS144603\_contig\_number\_15066SRS145308\_contig\_number\_2666SRS147271\_contig\_number\_13770SRS147346\_contig\_number\_66686SRS148091\_contig\_number\_14255SRS148511\_contig\_number\_21556SRS148721\_contig\_number\_55153SRS149879\_contig\_number\_24144SRS150029\_contig\_number\_5541SRS893230\_contig\_number\_11805SRS893270\_contig\_number\_21536SRS893279\_contig\_number\_14472SRS893292\_contig\_number\_8908SRS893295\_contig\_number\_12481SRS893300\_contig\_number\_11487SRS893380\_contig\_number\_127SRS893383\_contig\_number\_19375
821
species
100


SRS018888\_contig\_number\_contig-100\_39.56424SRS024492\_contig\_number\_16292SRS075821\_contig\_number\_17058SRS098571\_contig\_number\_32458SRS1041118\_contig\_number\_17042SRS144506\_contig\_number\_55157SRS147557\_contig\_number\_33702
species
7
2184759


SRS146764\_contig\_number\_7186
338188
1
species

2292935
3
species

SRS1055043\_contig\_number\_22884SRS1055067\_contig\_number\_10019SRS1055099\_contig\_number\_5125

46506
1
species

SRS149181\_contig\_number\_23245


SRS055966\_contig\_number\_675
1262750
species
1

1
family
1853231

1
genus
283168


SRS050925\_contig\_number\_4942
species
1
28118
